# Effect of Coal Rank and Coal Facies on Nanopore–Fracture Structure Heterogeneity in Middle-Rank Coal Reservoirs

**DOI:** 10.1021/acsomega.4c05179

**Published:** 2024-07-16

**Authors:** Cangyan Xiao, Donglin Han, Junjian Zhang, Shuzhao Chen, Zhenyuan Qin, Veerle Vandeginste

**Affiliations:** † School of Transportation Engineering, Jiangsu Vocational Institute of Architectural Technology, Xuzhou 221116, China; ‡ College of Earth Sciences & Engineering, Shandong University of Science and Technology, Qingdao 266590, China; § School of Mines, China University of Mining and Technology, Xuzhou 221116, China; ∥ Department of Mechanical, Materials and Manufacturing Engineering, Faculty of Engineering, University of Nottingham, Nottingham NG7 2RD, United Kingdom; ⊥ KU Leuven, Campus Bruges, Department of Materials Engineering, Bruges 8200, Belgium

## Abstract

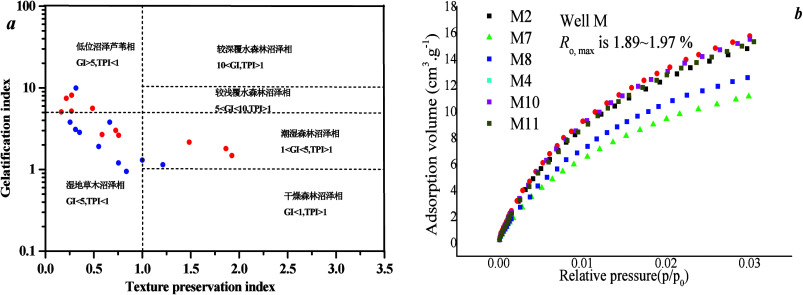

Considerable variations in microscopic and industrial components (ash, moisture, volatile matter, etc.) have been reported within identical coal seams. These disparities in coal quality and pore structure within the same coal seam profoundly affect the drainage of deep coalbed methane (DCBM). This study focuses on 22 coal samples collected from two wells in the Benxi Formation of the central and eastern parts of the Ordos Basin. First, the coal facies were determined for all samples using submicroscopic components, and then, the adsorption pore and seepage pore structures were studied through CO_2_/N_2_ adsorption and mercury intrusive tests. Subsequently, the study delves into the correlation between coal rank, coal facies, and the distribution of the pore structures across various pore sizes, elucidating the primary controlling factors influenced by coal rank and coal phase. The results are as follows: (1) For a given coal seam, *R*_o, max_ exhibits minimal variation among the samples, which suggests *R*_o, max_ is not the primary factor affecting pore structure. Conversely, the ash content occupies the pore space, thereby revealing a negative correlation between the ash content and adsorption pore volume (PV). (2) On the basis of the texture preservation index (TPI) and gelatification index (GI), coal facies were classified into moist forest swamp facies (type A), moist herbaceous swamp facies (type B), and water-covered herbaceous swamp facies (type C). Type A is characterized by higher TPI, lower GI, and ash content, whereas type C exhibits lower TPI, higher GI, and ash content. (3) Type A samples, with the lowest ash content, display larger PV and specific surface area (SSA) compared with type B, while type C has the lowest values. Type C, with the highest vitrinite content, predominantly consists of semibright and bright coal, prone to microcracks, which results in a higher seepage PV compared with types A and B. (4) The coal facies represent variations in ash content and microscopic components, which significantly impacts both adsorption and seepage pores. Moist forest swamp facies samples are characterized by micropore development and the highest content of adsorbed gas. Herbaceous swamp facies samples display macropore development and the highest content of free gas.

## 1 Introduction

Deep coalbed methane (DCBM) reservoirs exhibit favorable conditions, such as the larger thickness of coal seam, gas content, and storage scale; complete coal body structure; and weak hydrodynamic conditions.^1−4^ The higher gas saturation, pressure of the reservoir, and critical desorption pressure in these reservoirs lead to faster gas breakthrough and higher initial production. Notably, since 2022, the No. 8 coal seam in multiple DCBM wells in Daning-Jixian has achieved gas production exceeding 10^4^ m^3^ d^–1^ after segmented volume transformation, which marks a significant milestone in China’s deep coalbed methane drainage.^5^ Geological conditions for deep coalbed methane indicate that the thickness of deep coal reservoirs can exceed 10 m with significant differences across their vertical profile in terms of parameters, such as microscopic and industrial components, within the same seam of coal.^6,7^ This heterogeneity arises from continuous influences during the sedimentation process of peat, which introduces variability into the coal reservoir. The coal reservoir of vertical heterogeneity of coal reservoir pore structure has a significant effect,^8−10^ thereby making the exploration of the vertical variation pattern of pore–fracture structures in coal reservoirs crucial for achieving breakthroughs in DCBM production capacity.

Previous studies have delved into the variations in pore and fracture structures influenced by coal facies.^10−13^ Coal facies reflect the environmental geological factors, such as the hydrodynamic environment condition and oxygen content in water. Given their significant impact on coal seams’ gas-bearing capacity and pore formation, coal facies indices can effectively characterize the coal-forming swamp environment.^8^ These indices include the gelatinization index (GI) and texture preservation index (TPI) used to identify coal-forming bog environments, the transportation index (TI) for characterizing water body activity intensity, the groundwater flow index (GWI) reflecting overburden water depth, the vegetation index (VI) representing the relative source ratio of woody and herbaceous materials, and the mirror inertness ratio (V/I) used to determine the degree of oxidation of peat flats.^14−16^ Building on this foundation, Zhang et al.^9^ established four paleoenvironmental types, that is, wet forest swamp, intergradation forest swamp, drained forest swamp, and freshwater peat swamp, differentiated by rock type and maceral analysis. The study shows that the development of seepage pores is controlled by the coal phase under similar coalification conditions. Lou et al.^17^ indicate that the shallow-water-covered forest peat swamp facies exhibit higher porosity and larger pore size, thereby emphasizing the coal facies’ control of a key role in the reservoir pore structure of coal, which, in this study, influences of pore size distribution more clearly than coal rank. Zhao et al.^10^ reported a bimodal pattern in the pore structure of the wetland forest swamp facies in the eastern Ordos basin featuring well-developed micropores and poorly developed macropores.

Current literature has extensively explored the relationship between the pore structure and coal facies. However, few studies have investigated coal facies, adsorption pores, and seepage pore collectively. Most of the research has focused on examining the connection between the pore diameter at a certain stage and pore structure and coal facies. Importantly, the higher specific surface area (SSA) of adsorption pores (with a diameter less than 100 nm) is related to methane adsorption and desorption, which is influenced by the adsorbed methane content in deep coal reservoirs.^18−22^ Simultaneously, the exceptionally high pore volume (PV) of seepage pores (with a diameter greater than 100 nm) is associated with the seepage process, which is affected by the free methane content in deep coal reservoirs.^23−26^ Therefore, exploring the variation in pore structure between adsorption pores and seepage pores under the effect of coal facies holds significant importance. Meanwhile, previous research has established that maximum vitrinite reflectance (*R*_o, max_) is a crucial indicator characterizing the degree of coal evolution, which constrains the evolution process of pore and fracture system structures.^27−29^ For the same region, the change of pore and fracture systems under the combined constraints of *R*_o, max_ and the sedimentary environment (coal facies) requires thorough examination.

In this research, 22 samples of coal from two wells were collected from the Benxi Formation of the central and eastern Ordos Basin. The coal facies of all samples were classified on the basis of the texture preservation index (TPI) and gelatification index (GI). First, the coal facies of all samples were determined using submicroscopic components, and the adsorption pore and seepage pore structures were examined through CO_2_/N_2_ adsorption and mercury intrusive tests. Then, the correlation between coal rank, coal facies, and adsorption/seepage pore structures was discussed, explaining the main controlling pore sizes influenced by coal rank and coal facies.

## 2 Sampling Area and Experimental Method

### 2.1 Geological Setting and Sampling Collection

The study area is located in the eastern margin of the Ordos Basin, an extensive cratonic basin surrounded by mountains on all sides covering an area of 371 000 km^2^ and exhibiting a total thickness of 5000–10 000 m.^30−33^ In the study area, the Benxi Formation and Shanxi Formation are the main coal-bearing strata. The Benxi Formation’s coal-bearing section corresponds to the material source transportation and sedimentation stage, which forms a mixed sedimentary system comprising tidal flat, lagoon, barrier island, and swamp environments. In terms of its structure, the study area is situated in the middle of the Yishan slope and Jinxi fold belt. The basement of the Yishan Slope exhibits relatively minor undulations with a gentle distribution of overlying layers.^10^ The overall structure manifests as a gentle monocline dipping from the east to the west. The depth of the No. 8 coal seam ranges from 2400 to 2600 m, and its thickness varies from 6 to 15 m, averaging 9.3 m. From the M172 well and Qi 85 well, 23 coal samples were collected spanning the well from bottom to top.

According to the Chinese National standard GB/T 19222-2003,^34^ the collected samples were packaged using specific procedures and quickly transported to the laboratory for pretesting and a series of experimental tests. According to the Chinese national standard GB/T 6948-1998,^35^ the microstructure analysis of a 3 × 3 cm^2^ polished slab was carried out, and 500 points were analyzed for each sample. In addition, 10 samples were subjected to industrial analysis according to the national standard GB/T 212-2001.^36^

### 2.2 Experimental Methods and Calculation of Coal Facies

Considering the distinct impact of various pore sizes on coalbed methane drainage, the pore–fracture system can be categorized into adsorption pores (<100 nm) and seepage pores (>100 nm).^37^ Adsorption pores are further classified into micropores (<2 nm) and mesopores (2–100 nm) according to IUPAC guidelines, thereby resulting in a division of the pore–fracture system into micropores (<2 nm), mesopores (2–100 nm), and seepage pores (>100 nm).^25^

#### 2.2.1 High-Pressure Mercury Intrusion Test (HPMI)

The most commonly used method to analyze the seepage pore structure of coal reservoirs is the HPMI method. It determines essential information, such as the porosity, pore structure, pore connectivity, and pore compression coefficient, of coal. This test overcomes capillary forces by gradually increasing the pressure of the mercury injection. The maximum mercury inlet pressure for this test is 14.7 MPa, which covers a test pore size range of 3–10 000 nm.^38,39^

#### 2.2.2 Low-Temperature Carbon Dioxide/Nitrogen Adsorption Test (LTCO_2_/N_2_ GA)

In this test, 20 g of each sample is selected and ground to a particle size of 40–60 mesh. LTCO_2_/N_2_ GA is the prevailing method for analyzing the adsorption pore structure of coal reservoirs by providing insights into parameters, such as porosity, pore structure, and pore connectivity. Trostar III 3020 surface area and pore size distribution analyzer were used to detect the surface morphology of adsorption pores at 77 K. The PV and SSA of mesopores (2–100 nm) are determined using the Barrett–Joyner–Helenda (BJH) model,^40−43^ whereas the PV and SSA of micropores (<2 nm) are determined using the density functional theory (DFT) model.

#### 2.2.3 Coal Facies

The gel index (GI), tissue preservation index (TPI), and the GI-TPI phase diagram are widely used to identify coal facies, as proposed by Diessel.^9−13^
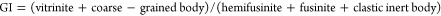
1

It represents the ratio of gel products to nongel products as an indicator of changes in water level and the degree of gel formation of plant remains in ancient peat swamps. A smaller GI value indicates relative dryness, whereas a larger GI value suggests the relative wetness of peat bogs.
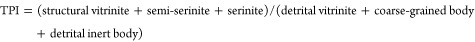
2

This index represents the ratio of structural to unstructured components in vitrinite and inert components; it reflects the degree of intact preservation of plant cell structure and the degradation intensity of plant tissue. A high TPI value signifies intact preservation of the plant cell structure with low degradation intensity with poor structural preservation. Various GI and TPI values correspond to eight distinct sedimentary environments, including lacustrine swamp, freshwater reed swamp, reed swamp, lowland swamp, wetland swamp (peat swamp), moist forest swamp, dry forest swamp, and land.

Groundwater impact index (GWI) indicates the degree of groundwater control over coal peat swamps, mineral content, and changes in the groundwater level. The boundary between forest swamps and flowing swamps is defined by GWI values of 1.
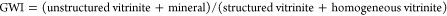
3

## 3 Results and Discussion

### 3.1 Coal Quality and Pore Type

**Figure 1:**
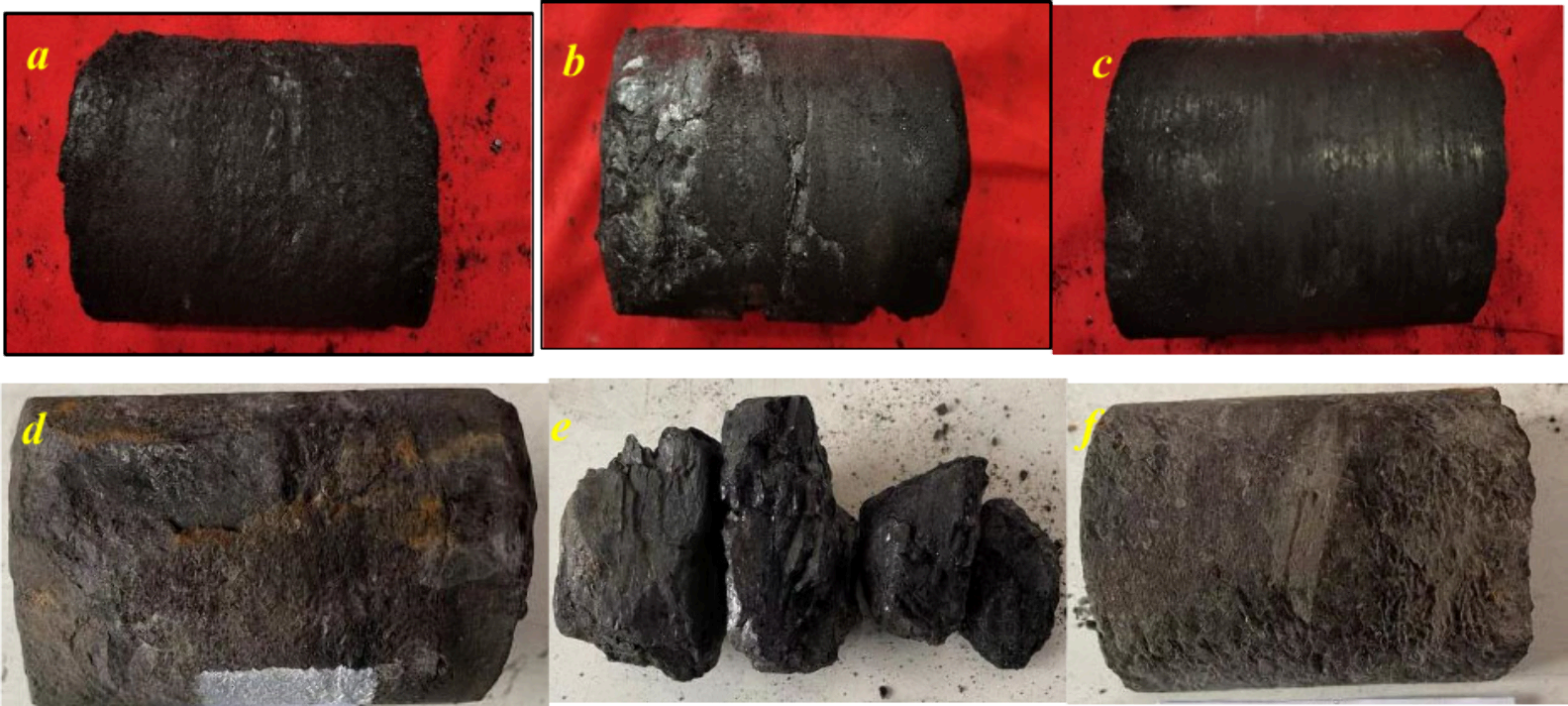
Typical macroscopic coal rock types and coal body structures. (a) Sample M1 semidull coal and primary structure coal. (b) Sample M4 semidull coal and primary structure coal. (c) Sample M7 semidull coal and primary structure coal. (d–f) Samples Q5, Q9, and Q11 as semibright coal and fragmented and mylonite coal.

The color of the No. 8 coal seam in the Benxi Formation of two wells appears as black and as stripe colors ranging from brown to brownish black (Figure 1). The coal seam structure exhibits a striplike, linear, and uniform pattern with stepped and uneven shapes, well-developed fractures, and containing thin layers of star-shaped pyrite. By comparing samples from well M172 and Q85, the coal from well M172 displays a significantly darker color than that from well Q85. Additionally, some coal samples from well Q85 exhibit fragmentation (Figure 1e), which indicates previous significant tectonic activity in the area. Macroscopically, the coal rock types from well M172 are mainly bright and semibright, whereas those from well Q8 are mainly semidull. Overall, the coal quality of well M1 is higher than that of well Q8. Moreover, the vitrinite reflectance *R*_o, max_ in this area gradually increases from the north to the south, ranging from 1.8 to 2.2%, which indicates a medium to high coal rank.

**Figure 2:**
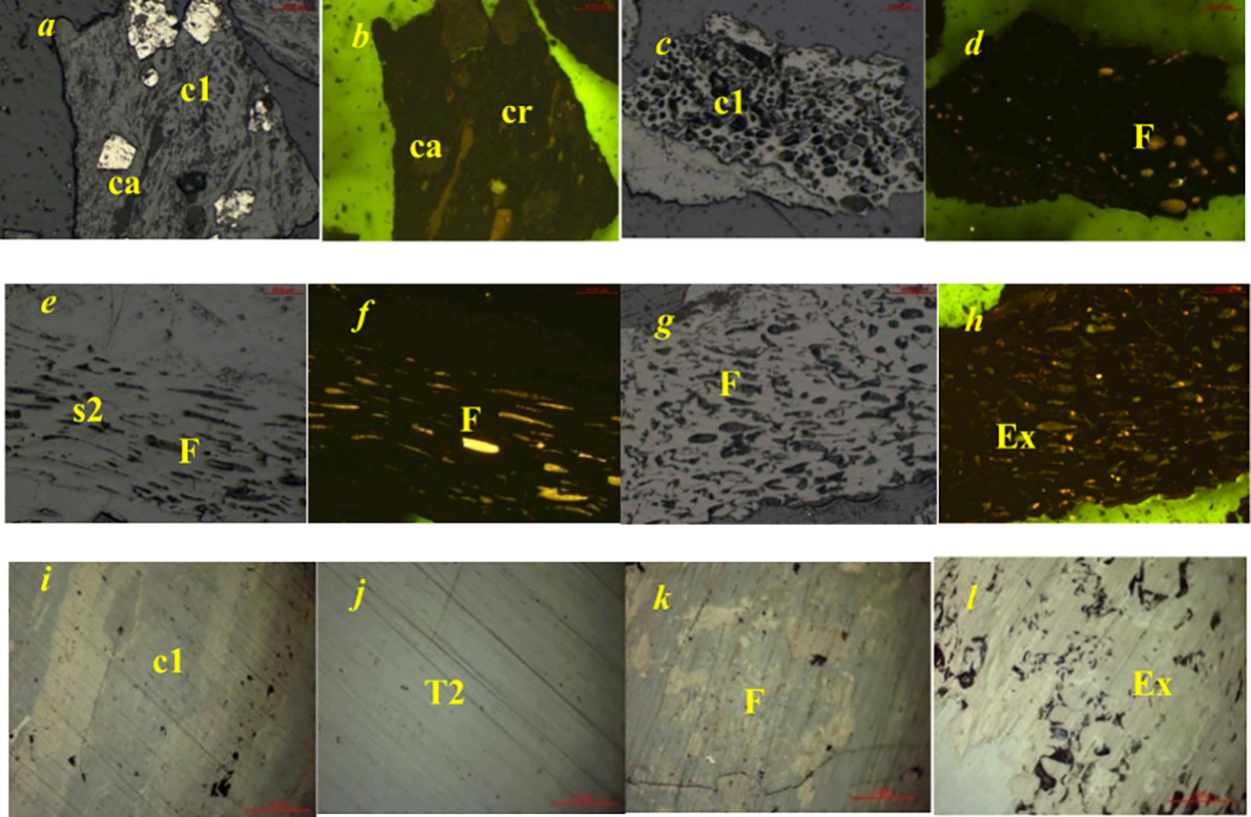
Microscopic features of submicroscopic components. (a) Sample M2: matrix vitrinite (c1) binds coarse-grained bodies, semifilamentous bodies, crustaceous bodies (cr), and minerals (ca) (b) Sample M2: Under the same field of view, minerals exhibit light green fluorescence, while keratinocytes, pollen bodies, and resin bodies exhibit orange yellow fluorescence. (c,d) Sample M3: Matrix vitrinite and serinite (F). (e) Sample M3: Filling and exuding asphaltene in the cell cavities of structural endosomes 2 (S2) and filamentous bodies (F) (f) Sample M3: The cavities of structural endosomes 2 and filamentous bodies are filled with exuding asphaltene, which emits orange yellow fluorescence (F). (g,h) Sample M10: Silklike body (F) and well-preserved cell cavity filled with exuded asphalt (EX) and clay minerals. (k–n) Sample Q10: Matrix vitrinite, homogeneous vitrinite, cementitious serinite fragments and detrital inertinite, semiserinite).

The coal macerals of the No. 8 coal seam are mainly composed of vitrinite followed by inertinite (Figure 2). Vitrinite consists mostly of matrix vitrinite followed by structural vitrinite, homogeneous environment vitrinite, and a small amount of clustered vitrinite (Figure 2). Inertinite components predominantly include semifilamentous bodies with a minor presence of filamentous bodies and almost no coarse-grained or detrital inertinite bodies. The shell composition primarily comprises spore pollen with a small amount of keratin. Vitrinite is the main microscopic component of the coal, which appears gray under reflected light irradiation. Inertinite components commonly exhibit protrusions under reflected light and appear white. Comparing samples from well M172 and Q85, the vitrinite content of coal samples from well M1 ranges from 43.5 to 65.54% (average of 53.43%), which is lower than that of well Q1 (ranging from 48.83 to 93.34, with an average of 63.03%). However, the liptinite content of coal samples from well M1 ranges from 1.69 to 18.54% (average of 10.59%), which is higher than that of well Q1 (ranging from 0.34 to 2.13, with an average of 0.97%).

**Figure 3:**
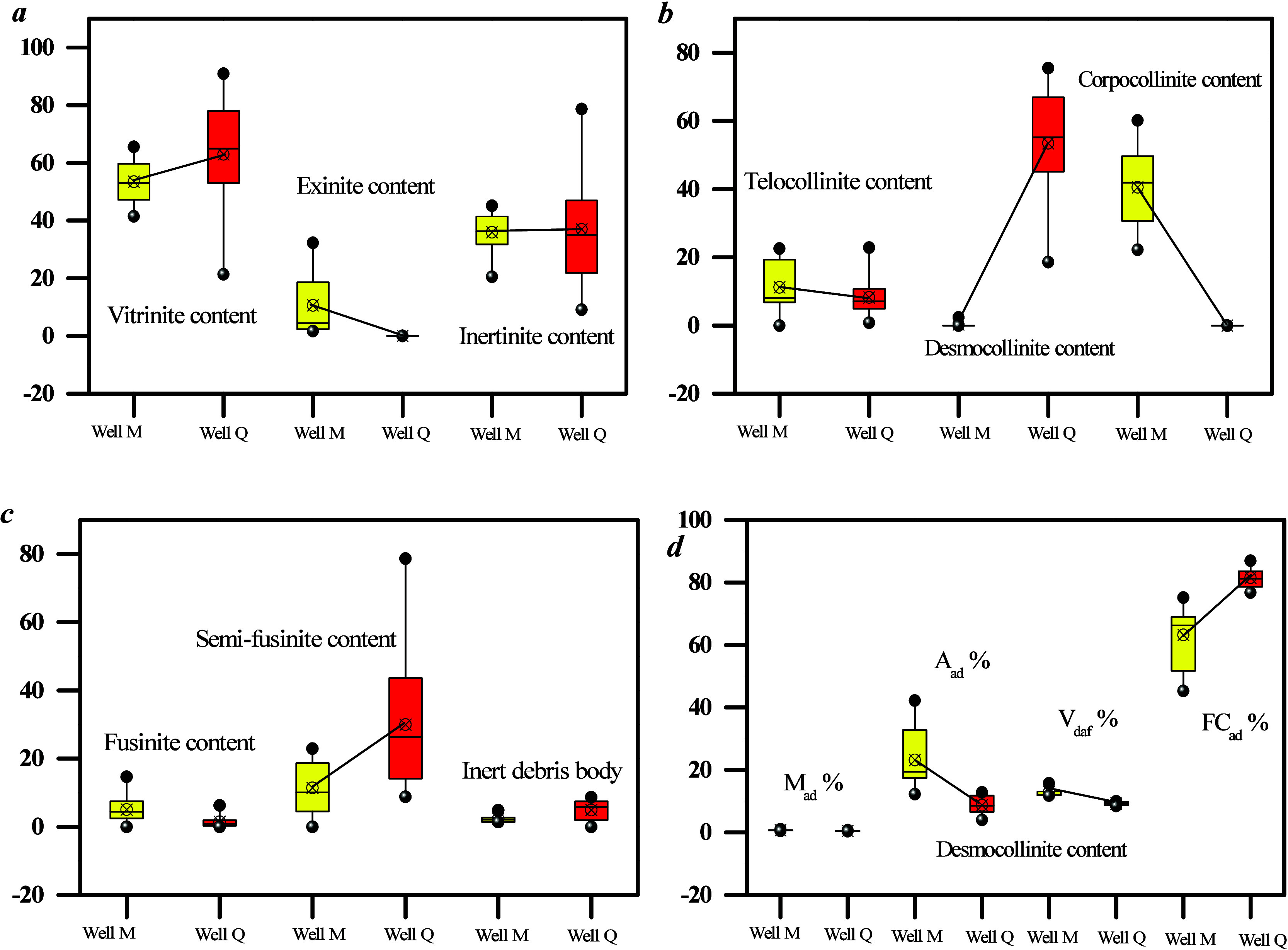
Content of macerals, submacerals, and industrial components in coal samples collected from two wells.

The depth of coal samples collected from well Q1 (2627–2635 m) is larger than those collected from well M1, which results in a higher *R*_o, max_ for well Q1 (1.97–2.26%) compared with well M1 (1.87–1.98%). As coalification progresses, the vitrinite content gradually increases, which leads to a higher vitrinite content in the coal samples of well Q1 (Figure 3a). Figure 5b shows that the desmocollinite content of well Q1 is higher than that of well M1, and the desmocollinite content significantly impacts the hydrocarbon generation potential (Figure 3b), thereby indicating a stronger hydrocarbon generation potential in well Q1 compared with well M1. Moreover, Figure 3c,d illustrates that the ash content of well M1 is higher than that of well Q1, which suggests stronger hydrodynamic conditions during peat accumulation in well M1, thereby leading to an increase in the foreign mineral content. This underscores the strong water flow activity in the coal-forming environment of well M1.

### 3.2 Effect of Coal Rank on Pore Distribution Heterogeneity

**Figure 4:**
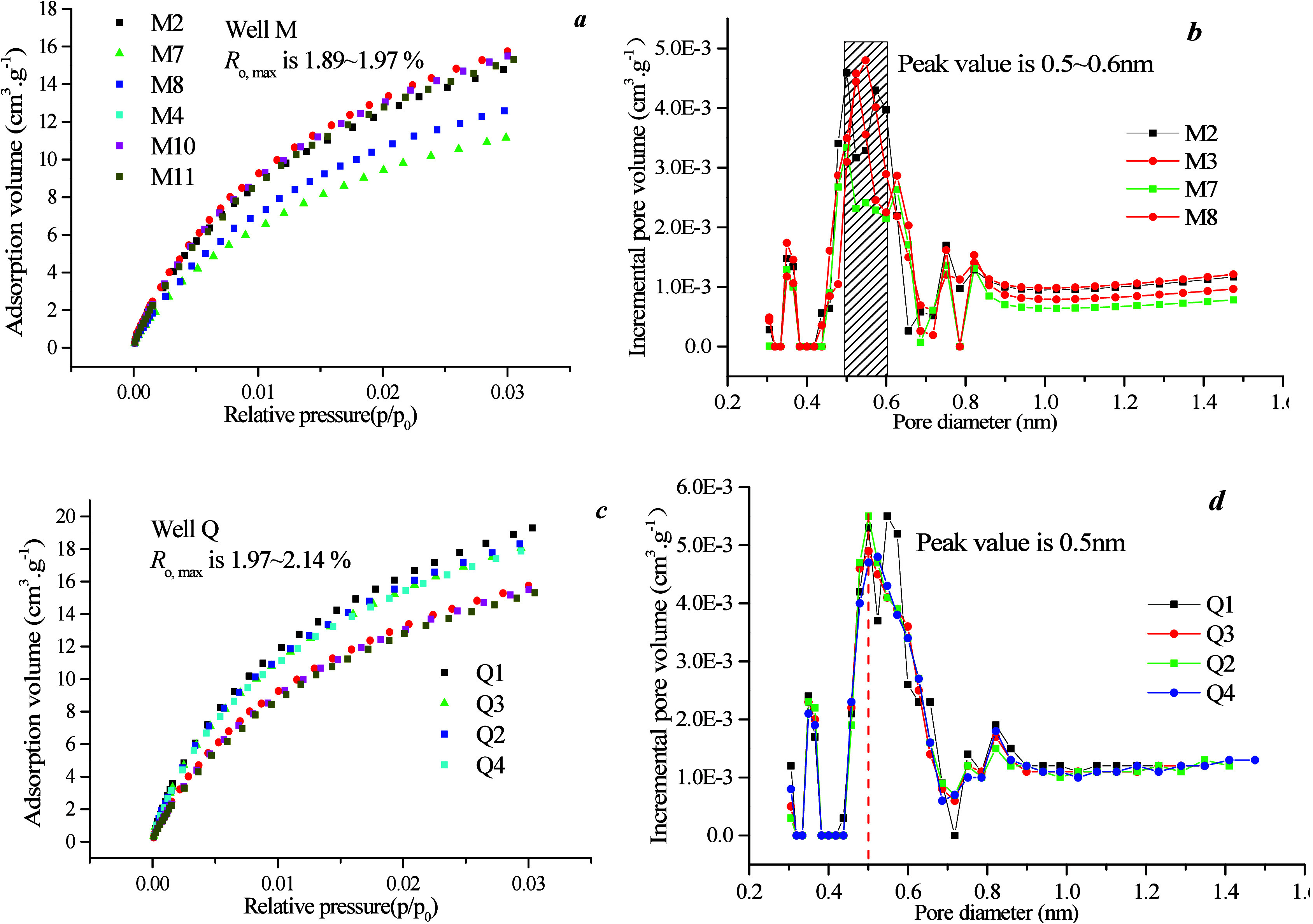
Carbon dioxide adsorption curves and pore size distribution of different coal rank: (a,c) carbon dioxide adsorption curves, and (b,d) micropore diameter distribution.

Micropore distribution was analyzed using LTCO_2_ GA (Figure 4). Figure 6a illustrates that the maximum CO_2_ adsorption capacity of well M ranges from 10 to 16 cm^3^ g^–1^, with an average of 3.24 cm^3^ g^–1^, which is lower than that of well Q (having a maximum CO_2_ adsorption capacity of 14–20 cm^3^ g^–1^ with an average of 18.14 cm^3^ g^–1^) (Figure 4a,c). This discrepancy can be attributed to the higher *R*_o, max_ leading to an increase in micropores, and subsequently, total SSA. Figure 4b,d shows the distribution curve of micropores, which displays a three-peak pattern, indicating that the PV and SSA within the range of 0.5–0.6 nm contribute significantly to the volume of the coal sample and its surface area.

**Figure 5:**
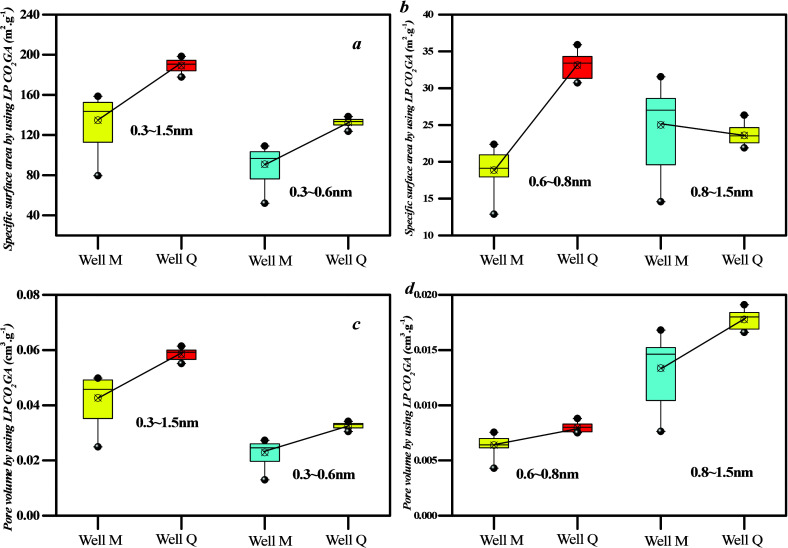
Different coal rank coal samples’ meso-PV and SSA: (a,b) specific surface area and (c,d) pore volume.

On the basis of the data presented in Figure 4b,d, micropores can be divided into 0.3–0.6, 0.6–0.8, and 0.8–1.5 nm. The PV and specific area of 0.3–0.8 nm pores from well Q are higher than those from well M (Figure 5a), whereas the PV and specific area of 0.8–1.5 nm pores are similar between well Q and well M (Figure 5b). This observation is due to the fact that the PV and specific area percentage of 0.3–0.8 nm pores range from 80 to 85%, which indicates that this range of pore diameter contributes significantly to the SSA. Conversely, the PV and specific area percentage of 0.8–1.5 nm pores range from 15 to 20%, which thereby indicating a smaller proportion of the SSA. Moreover, there is a good linear positive correlation between the SSA and volume of micropores resulting in higher PV and specific area of 0.3–1.5 nm pores in samples from well Q compared with well M.

**Figure 6:**
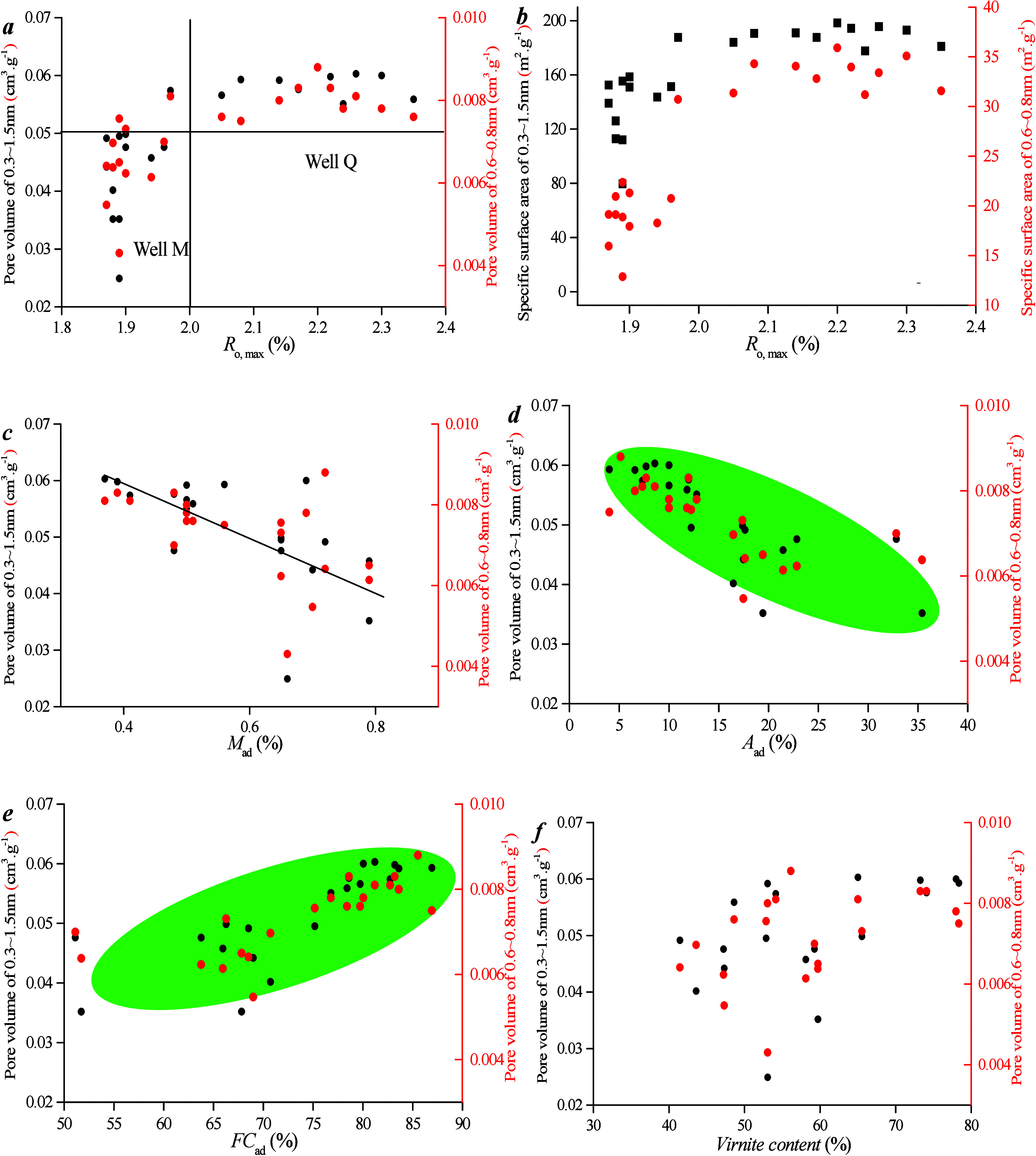
Correlation between microporous PV, maceral content, and industrial components: (a) *R*_o, max_ vs pore volume of micropore, (b) *R*_o, max_ vs specific surface area of micropore, (c) *M*_ad_ vs pore volume, (d) *A*_ad_ vs pore volume, (e) *F*_Cad_ vs pore volume, and (f) Vitrinite content vs pore volume.

Correlations among microporous PV, maceral content, and industrial components are studied (Figure 6). As can be seen from Figure 8a,b, there is no obvious linear relationship between *R*_o, max_ and PV/SSA. This is attributed to the fact that all samples are obtained from the same coal seam with minimal variation in vertical depth within the seam. Consequently, the slight differences in *R*_o, max_ among coal samples from the same well do not significantly impact the micropore development characteristics across different depths within the same well. However, it is noteworthy that PV and specific area of 0.3–1.5 nm pores in samples from well Q are higher than those from well M, likely due to the greater depth of coal samples collected from well Q1 (2627–2635 m) compared with those from well M1, which results in a higher *R*_o, max_ for well Q1 (1.97–2.26%) compared with well M1 (1.87–1.98%).

As can be seen from Figure 8c–f, there is no obvious linear relationship between vitrinite content and PV/SSA. This lack of correlation stems from the fact that vitrinite content is primarily influenced by *R*_o, max_. Conversely, PV and SSA of micropores increase as ash content decreases and fixed carbon content increases. A higher ash content can clog micropores. In conclusion, within the same coal seam, the variation in *R*_o, max_ among samples is minimal, which renders it a less significant factor affecting micropore structure. However, the ash content can occupy pore space, thereby resulting in a negative correlation between the ash content and micro-PV.

**Figure 7:**
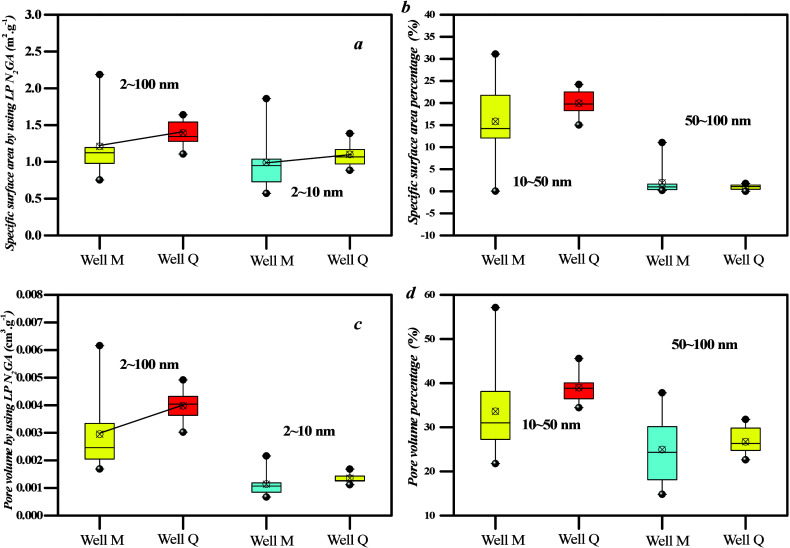
Different coal rank coal samples’ meso-PV and SSA: (a,b) specific surface area and (c,d) pore volume.

**Figure 8:**
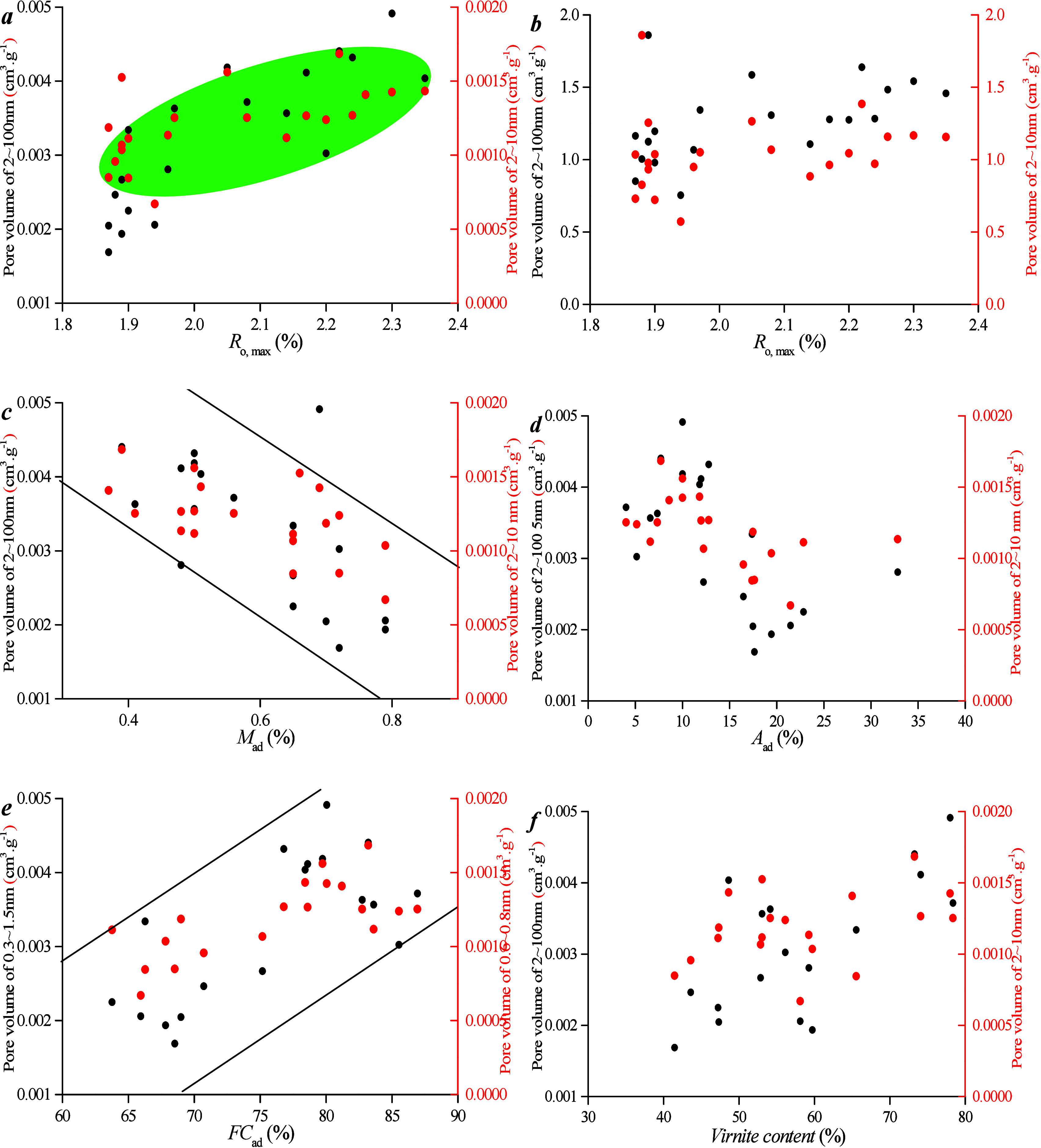
Correlation between meso-PV and maceral content and industrial components for different coal ranks: (a) *R*_o, max_ vs pore volume of micropore, (b) *R*_o, max_ vs specific surface area of micropore, (c) *M*_ad_ vs pore volume, (d) *A*_ad_ vs pore volume, (e) *FC*_ad_ vs pore volume, and (f) vitrinite content vs pore volume.

**Figure 9:**
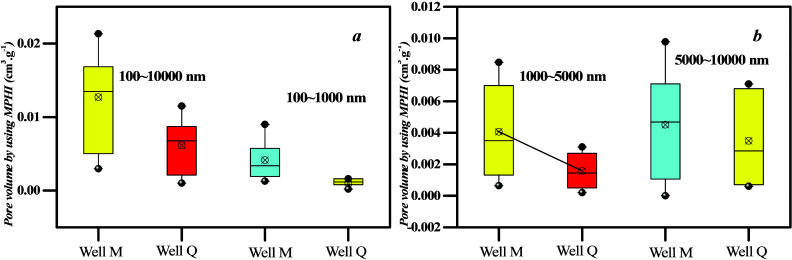
Different coal rank coal samples’ meso-PV and SSA.

**Figure 10:**
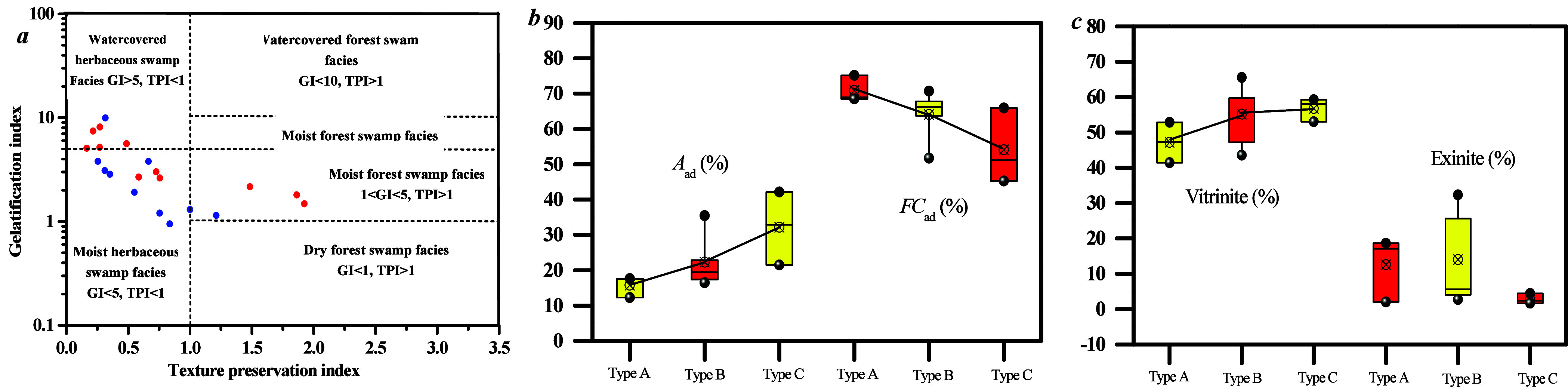
Classification of coal facies on the basis of several parameters and microscopic components/coal parameters for different coal facies types: (a) GI–TPI, (b) VI–GWI, (c) *A*_ad_–*FC*_ad_, and (d) vitrinite–liptinite.

The distribution of mesopores was analyzed using LTN_2_ GA (Figure 7). The results reveal a distribution curve with a single peak pattern, which indicates that the PV and SSA of 2–10 nm pores constitute a significant portion of the volume and SSA of coal samples. However, the heterogeneity in mesopore surface area of well Q1 appears weaker than that of well M1. On the basis of Figure 9, mesopores can be divided into 2–10, 10–50, and 50–100 nm pores. The PV and specific area of 2–100 nm pores in samples collected from well Q are higher than those from well M (Figure 7a), although the PV and specific area of 50–100 nm pores are similar between well Q and well M (Figure 7b–10d). This observation can be interpreted as the fact that the PV and specific area percentage of 2–10 nm pores range from 73 to 85%, thereby indicating a significant contribution to the SSA.

The relationship between mesoporous volume and maceral content, as well as industrial components, was investigated (Figure 8). Figure 8 a,b shows a positive relationship between *R*_o, max_ and SSA for 2–100 nm pores but without the positive relationship between *R*_o, max_ and PV for the pore range. This distinction from micropores stems from significant differences in the PV distribution and SSA of mesopores. Figure 8c–f illustrates no clear linear relationship between vitrinite content and PV/SSA. This is attributed to the fact that vitrinite content is primarily controlled by *R*_o, max_. In addition, the PV and SSA of micropores increase as fixed carbon content increases. However, there is not a positive relationship between ash content and PV of 2–100 nm pores. This discrepancy arises from the relatively smaller volume and specific surface percentage of mesopores, which results in a weaker correlation between ash content and PV/SSA. In conclusion, the development of mesopores is often influenced by the thermal evolution degree and industrial components.

Seepage pore distribution was determined using HPMI (Figure 9). The results show that when the mercury injection pressure is below 0.1 MPa, the amount of mercury injected into the coal sample from well Q1 increases sharply, whereas the increase in the amount of mercury injected into the coal sample from well M1 is gradual. This suggests that PV of nanopores in well Q1 is higher than that in well M1 (Figure 9 a,c). The higher *R*_o, max_ and stronger structural deformation strength are the main reasons for the increase of nanopore PV in coal samples collected from well Q1. Conversely, when the mercury injection pressure exceeds 0.1 MPa, the amount of mercury injected into the coal sample from well Q1 increases slowly, whereas the increase in the amount of mercury injected into the coal sample from well M1 is rapid. This shows that the PV of larger pores in well Q1 is lower than that in well M1 (Figure 9a,c). In addition, Figure 12b,d demonstrates that the pore size distribution heterogeneity of coal samples collected from well Q1 is smaller than that of well M1. However, the PV and SSA of coal samples collected from well M1 are larger than those from well Q1 (Figure 9).

Unlike micropores and mesopores, they do not correlate with seepage PV and maceral content or industrial components. This is because various factors affect the pore structure of the seepage pore. Previous studies suggest that the volume of macropores gradually decreases as the degree of thermal evolution increases. Nevertheless, Figure 1 shows that the macroscopic coal rock types of well M1 are mainly bright and semibright, which makes them prone to fracture formation during the coal formation process. For coal samples with developed microcracks, the effect of ash filling on PV is relatively weak.

### 3.3 Effect of Coal Facies on Pore Distribution Heterogeneity

On the basis of TPI–GI, three coal facies have been determined, namely, moist forest swamp facies (type A), moist herbaceous swamp facies (type B), and covered herbaceous swamp facies (type C) (Figure 10a,b). Type A is characterized by TPI > 1 and GI < 1, type B by TPI < 1 and GI < 5, and type C by TPI < 1 and GI > 5. The plant cell structure in the moist forest swamp facies is well preserved with lower degradation intensity. In addition, Figures 15c,d reveals that the ash content of class C is higher than that of class B, with class A having the lowest ash content. The moist forest swamp environment, characterized by shallow water cover and weak hydrodynamic conditions, limits the transport of minerals into the coal-forming swamp, which results in a lower ash content. Conversely, the water-covered herbaceous swamp with deep water coverage and strong hydrodynamic conditions allows for the transport of a greater amount of minerals into the coal-forming swamp, thereby leading to a higher ash content. The depth of water coverage also influences oxygen levels with stronger reduction conditions increasing the vitrinite content (Figure 10d).

**Figure 11:**
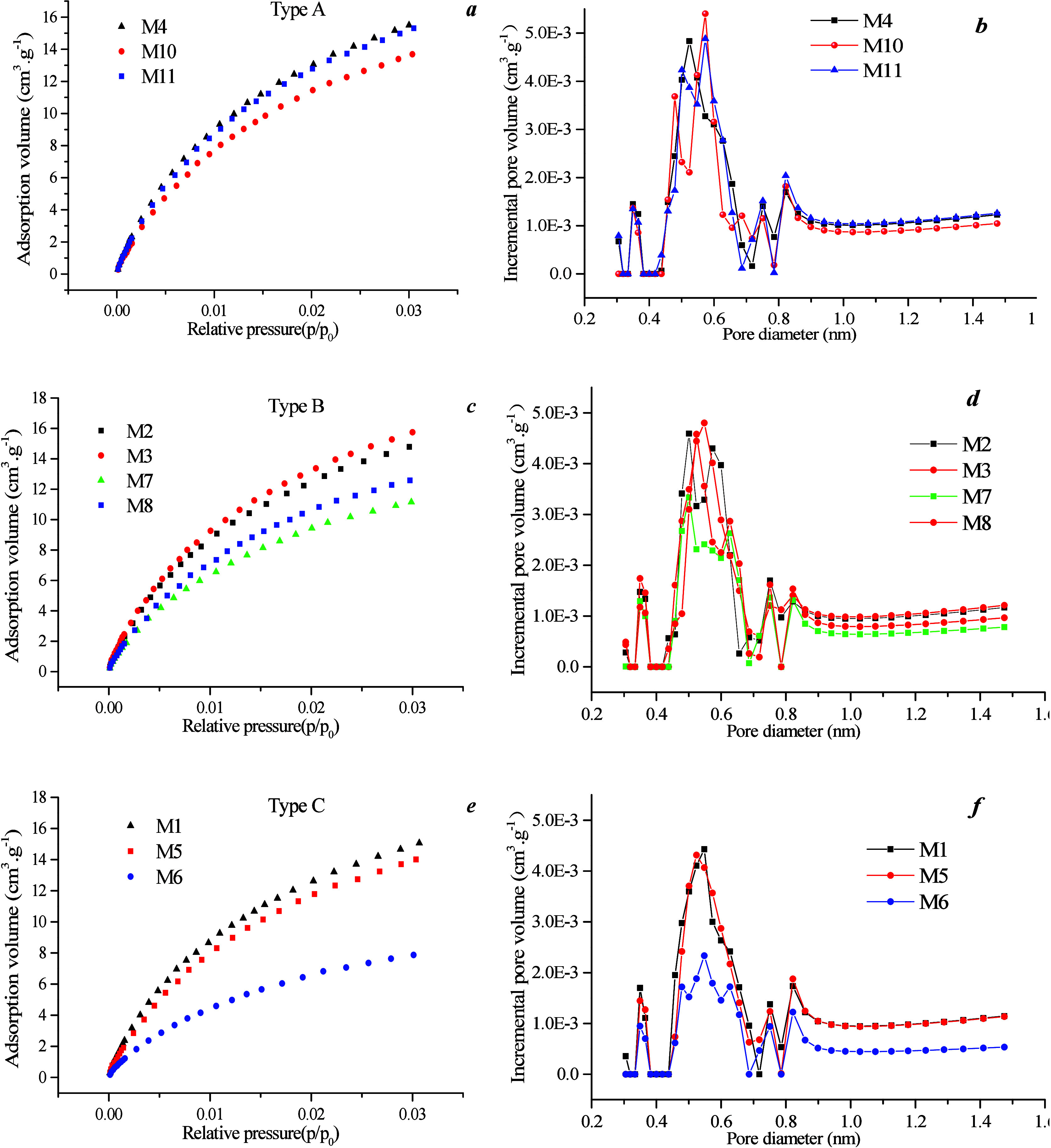
Distribution of carbon dioxide adsorption curve and pore size in different coal phases.

Figure 11a,c,e illustrates that the maximum CO_2_ adsorption capacity of type A is 14–16 cm^3^ g^–1^ with an average of 14.44 cm^3^ g^–1^, which is higher than that of type B (maximum CO_2_ adsorption capacity ranging from 10 to 14 cm^3^ g^–1^ with an average of 13.14 cm^3^ g^–1^), while type C exhibits the lowest maximum CO_2_ adsorption capacity. This is attributed to higher ash content leading to a decrease in micropores, which consequently reduces the total SSA. Furthermore, Figure 11b,d,f shows a distribution curve of micropores with a three-peak pattern, thereby indicating that the PV and SSA of 0.5–0.6 nm pores contribute significantly to the volume and SSA of coal samples.

**Figure 12:**
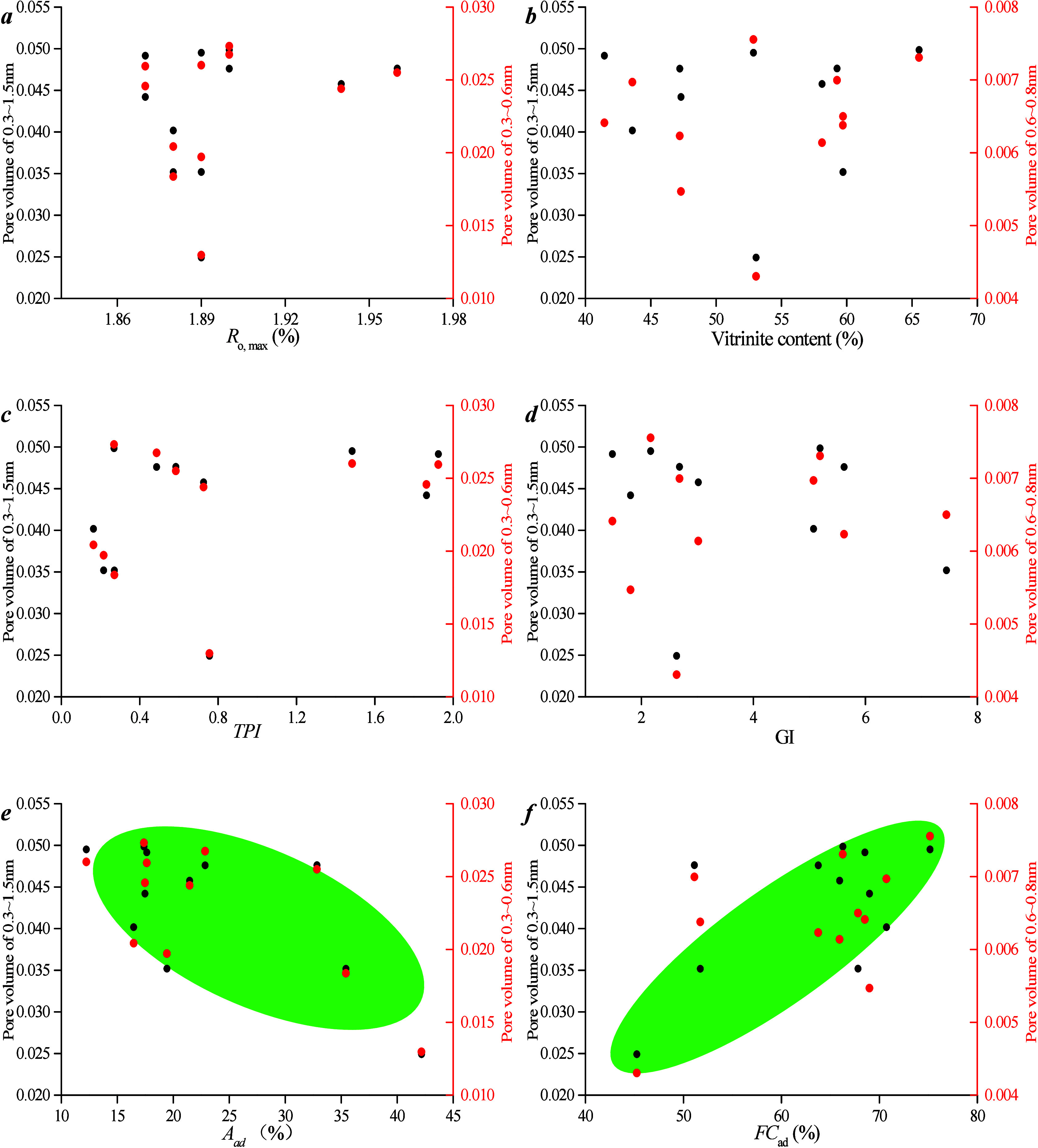
Correlation between microporous PV and coal facies parameters and industrial components: (a) *R*_o, max_ vs pore volume of micropore, (b) vitrinite content vs pore volume, (c) TPI vs pore volume, (d) GI vs pore volume, (e) *A*_ad_ vs pore volume, and (f) *FC*_ad_ vs pore volume.

Correlations between micro-PV and maceral content, as well as industrial components, were examined (Figure 12). Figure 12a,b demonstrates that there is no clear linear relationship between *R*_o, max_ and PV/SSA. This is attributed to the fact that all samples are collected from the same coal seam, and the differences in vertical depth within the same coal seam are minimal, which results in slight variations in *R*_o, max_ among coal samples at different depths within the same well. Consequently, the differences in micropore development characteristics among coal samples at different depths within the same well are not pronounced. Figure 12c–f shows that there is no obvious linear relationship between vitrinite content and PV/SSA. This is because the content of vitrinite is primarily controlled by *R*_o, max_. However, ash content can occupy pore space, which leads to a negative correlation between ash content and micro-PV.

**Figure 13:**
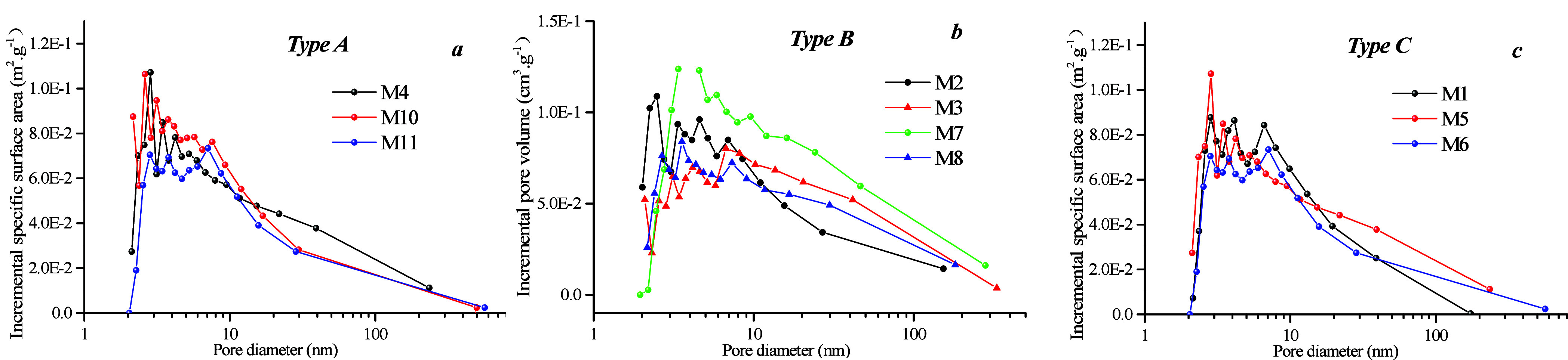
SSA of mesopores based on nitrogen adsorption–desorption curves for different coal facies.

**Figure 14:**
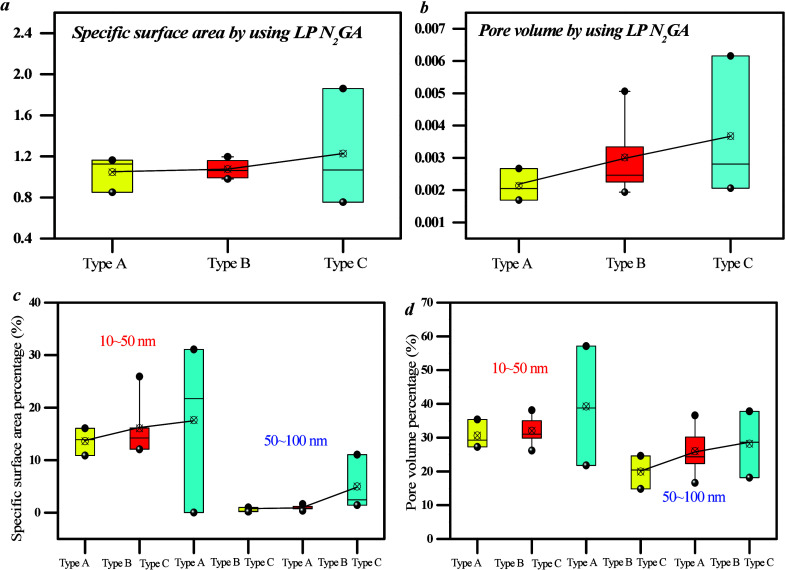
Meso-PV and SSA with different lithofacies samples

Mesopore distribution was analyzed using LTN_2_ GA (Figure 13). The results indicate that the distribution curve of mesopores exhibits a single peak pattern, thereby suggesting that the PV and SSA of 2–10 nm pores constitute a significant proportion of the volume and SSA of coal samples. However, the mesopore surface area heterogeneity of type A is weaker than that of types B and C. On the basis of Figure 13, mesopores can be divided into 2–10, 10–50, and 50–100 nm pores. The PV and specific area of 2–100 nm pores of type C are higher than those of type A (Figures 13 and 14), whereas the PV and specific area of 10–50 nm pores among the three coal facies are similar. This is because the PV and specific area percentage of 10–50 nm pores range from 10 to 23%, thereby indicating that these pores contribute a lower proportion of SSA.

**Figure 15:**
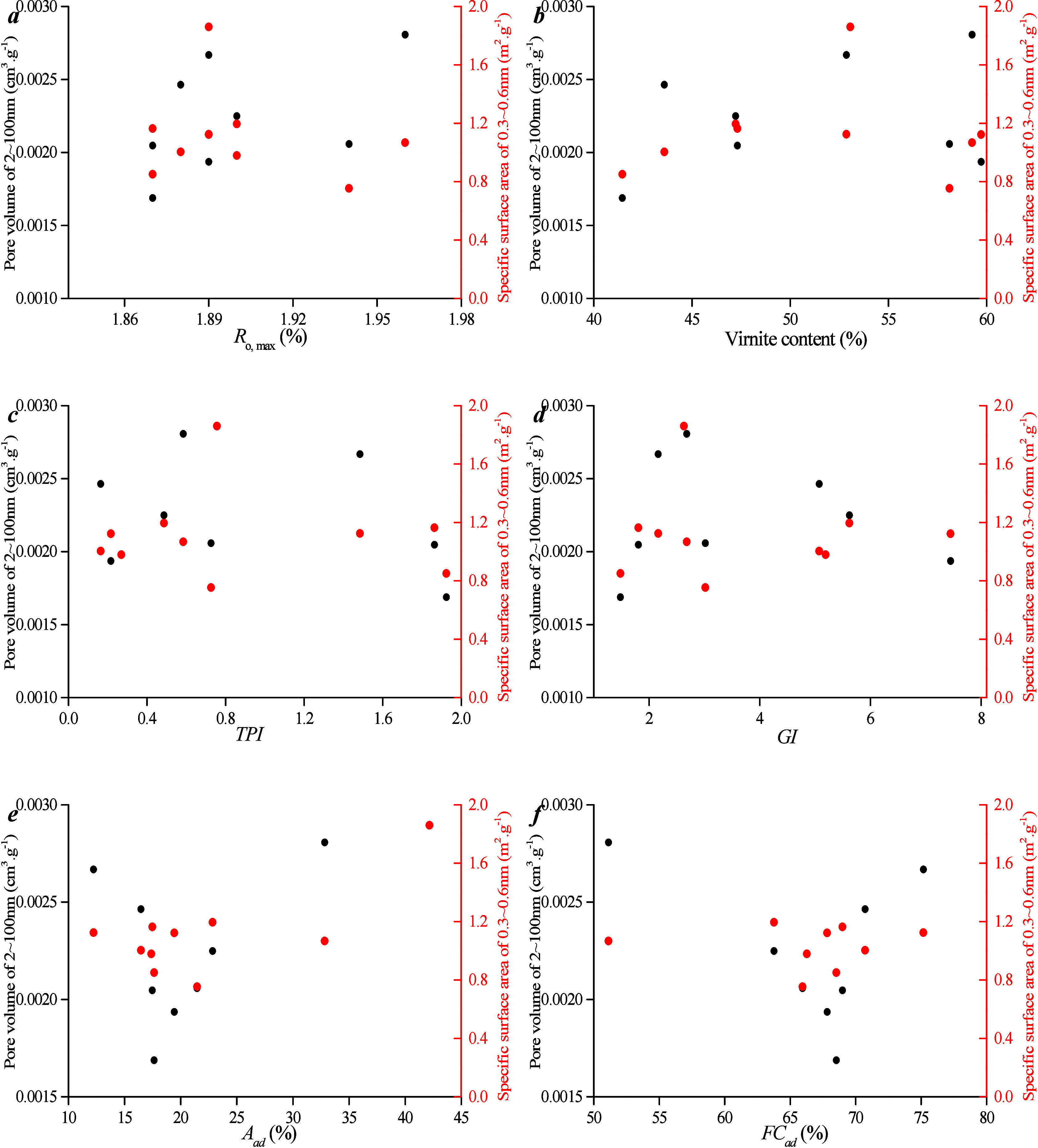
Correlation between meso-PV and coal facies parameters and industrial components: (a) *R*_o, max_ vs pore volume of micropore, (b) vitrinite content vs pore volume, (c) TPI vs pore volume, (d) GI vs pore volume, (e) *A*_ad_ vs pore volume, and (f) *FC*_ad_ vs pore volume.

Figure 15a,b shows that there is no clear relationship between *R*_o, max_ and SSA, nor for PV, for 2–100 nm pores. Figure 15c–f demonstrates that there is no apparent linear relationship between coal facies parameter/industrial components and PV/SSA. This indicates that the influence of 2–100 nm pores on coal facies is weaker.

## 4 Conclusions

This study focused on the analysis of 22 coal samples collected from the Benxi Formation of the central and eastern parts of the Ordos Basin. On the basis of the texture preservation index (TPI) and gelatification index (GI), the coal facies of all samples were identified. First, the coal facies were determined on the basis of submicroscopic components, and further investigations were conducted on the adsorption and seepage pore structures using CO_2_/N_2_ adsorption and mercury intrusion tests. Subsequently, correlations among coal rank, coal facies, and adsorption/seepage pore structures were explored, thereby elucidating the main controlling factors influenced by coal rank and coal facies. The key conclusions drawn from this study are as follows.

The ash content of well M1 is higher than that of well Q1, thereby indicating stronger hydrodynamic conditions during peat accumulation, which leads to an increased content of foreign minerals. This indicates greater water flow activity in the coal-forming environment in well M1.

For the same coal seam, *R*_o, max_ does not primarily dictate micropore structure. Instead, the ash content plays a significant role in occupying pore space, thereby exhibiting a negative correlation with micro-PV. Moreover, the development of mesopores is often controlled by the degree of thermal evolution and industrial components.

Higher ash content leads to a decrease in micropores, consequently decreasing the total surface area. As a result, the PV and specific area of 0.3–1.5 nm pores in type A are higher than those in types B and C, whereas the PV and specific area of 0.6–1.5 nm pores are similar across all three types.
